# Four-Stub Resonator-Coupled MIM Waveguide Sensor

**DOI:** 10.3390/s26061779

**Published:** 2026-03-11

**Authors:** Jifan Yang, Shubin Yan, Zhenyang Xu, Yang Cui, Youbo Hu, Guang Liu, Dengchang Ma, Taiquan Wu

**Affiliations:** 1 School of Electrical and Control Engineering, North University of China, Taiyuan 030051, China; 2 School of Electrical Engineering, Zhejiang University of Water Resources and Electric Power, Hangzhou 310018, China; 3 Joint Laboratory of Intelligent Equipment and System for Water Conservancy and Hydropower Safety Monitoring of Zhejiang Province and Belarus, Hangzhou 310018, China; 4 School of Geomatics Science and Technolog, Zhejiang University of Water Resources and Electric Power, Hangzhou 310018, China

**Keywords:** surface plasmon polaritons, fano resonance, nanosensor, temperature measurement

## Abstract

In this design, we propose a completely new sensor structure. It features a metal–insulator–metal (MIM) waveguide and a circular four-stub resonator (CFSR). Using the finite element method, we analyzed the performance of the sensor structure. We examined the impact of different parameters and structural variations on its performance. Ultimately, we determined the optimal performance parameters for the best configuration. The modified device demonstrated a sensitivity (S) of 2940 nm/RIU and a figure of merit (FOM) of 52.5. Furthermore, this sensor design demonstrates significant potential for temperature measurement applications, with a core parameter of 1.508 nm/°C.

## 1. Introduction

The surface plasmon polaritons exist at the interface between metals (or other conductive materials) and dielectrics (such as air or glass) [1,2,3]. SPPs are hybrid excitations formed by the coupling of light (photons) with the collective oscillation of free electrons (plasmons) on a metal surface. When light strikes the metal surface, it “shakes” the free electrons inside the metal. This causes the electrons to move collectively, like waves, propagating along the metal surface [4,5,6]. At the same time, the light itself becomes confined near the metal surface and does not propagate further. This “electron density wave” is the surface plasmon polariton.

SPPs possess the following unique properties: field enhancement, subwavelength confinement, and a specific dispersion relation [7,8]. These properties allow SPPs to overcome the diffraction limit of traditional optics. They also provide a way to manipulate subwavelength light waves on a two-dimensional plane.

As a result, SPPs are widely used in various fields. These include surface-enhanced spectroscopy [9,10], highly sensitive biochemical sensing [11,12], subwavelength photonics and integrated optical circuits [13,14], perfect absorbers and thermophotovoltaics [15,16], nanolithography and super-resolution imaging [17,18], as well as photothermal therapy and drug delivery [19].

Fano resonance is a special resonance phenomenon that arises from the constructive and destructive interference between a discrete state and a continuum of propagating modes [20,21,22]. This phenomenon typically occurs in coupled systems, such as a resonant cavity side-coupled to a waveguide, which provide the discrete and continuous channels, respectively.

Its most distinct signature in the frequency domain is an asymmetric and sharp spectral lineshape featuring a narrow resonance dip adjacent to a broad background. This unique lineshape confers two critical advantages for sensing [23,24]: first, the narrow linewidth implies a high quality factor and thus fine spectral resolution; second, the discrete state is highly sensitive to external perturbations, leading to a large spectral shift per unit change in the refractive index, i.e., high sensitivity. The combination of these features results in a high figure of merit, which is the key metric for refractive index sensors.

Because of these advantages, Fano resonance is widely used in optics and plasmonics to enhance light–matter interaction and improve device performance. In surface plasmon polariton systems, Fano resonance commonly arises from the interference between localized SPPs and propagating SPPs. The strong field confinement and tunable dispersion of SPPs offer an ideal platform for realizing and controlling pronounced Fano resonances.

The metal–insulator–metal (MIM) waveguide provides two parallel metal–dielectric interfaces [25,26,27]. These interfaces are placed very close to each other. The modes on these two interfaces couple strongly. This forms a new propagation mode, confined within the central insulator layer. This is called the gap SPP mode. In recent years, the design of refractive index sensors based on SPPs has witnessed significant advances. Zegaar et al. proposed a high-bandwidth plasma device (UWB-BSF) [28]. The structure of the waveguide is composed of a waveguide and a hexagonal cavity. This nanostructure functions within the mid-infrared range. The device under consideration features a wide stopband and tunable properties. The solution is advantageous for plasma-integrated circuits and transmission.

Wu Qiaohua et al. designed a device system based on waveguides and multiple ring resonators [29]. It enables independent tuning of dual resonances. Yunping Qi et al. proposed a triple-ring nested cavity structure [30]. This design breaks through the limitation of traditional structures, which lack dynamic tunability.

At the same time, Bound States in the Continuum (BICs), as non-radiating states embedded within the continuous spectrum of radiating modes, can greatly enhance and localize electromagnetic fields. Ideal BICs possess infinitely high quality (Q) factors. When the symmetry or momentum-matching condition of a BIC is broken, it degenerates into a quasi-BIC with a finite yet extremely high Q factor. Quasi-BICs provide a novel physical mechanism for realizing ultra-sensitive sensing. Researchers have achieved quasi-BICs in various structures, including photonic crystal slabs, metasurfaces, and resonant grating waveguide structures. In particular, the momentum-mismatch-driven quasi-BIC realized in resonant grating waveguide structures has attracted attention due to its relatively simple structure and ease of fabrication. Leveraging the ultrahigh-Q resonance of quasi-BICs not only enables significant enhancement of the Goos-Hänchen (GH) shift but also opens up new avenues for designing ultra-sensitive sensors. For example, based on the quasi-BIC-enhanced GH shift, a temperature sensor with a resolution on the order of 10^−4^ °C was designed. Similarly, utilizing the ultrahigh sensitivity of quasi-BICs, high-precision optical detection of hemoglobin concentration has been achieved, with a resolution reaching the order of 10^−3^ g/L [31,32,33,34].

## 2. Model Design and Analysis

This paper proposes a novel coupling structure based on an MIM waveguide. The design is composed of a MIM waveguide and a circular four-short stub resonator (CFSR). Verification and simulation were conducted using the finite element method (FEM). Simulation tests show that this structure significantly enhances the system’s sensitivity (S). It also reduces the bandwidth. Furthermore, the influence of several structural parameters on performance was investigated. These parameters include the outer radius of the CFSR, the number of stubs in the CFSR, and the length of the stubs.

**Figure 1:**
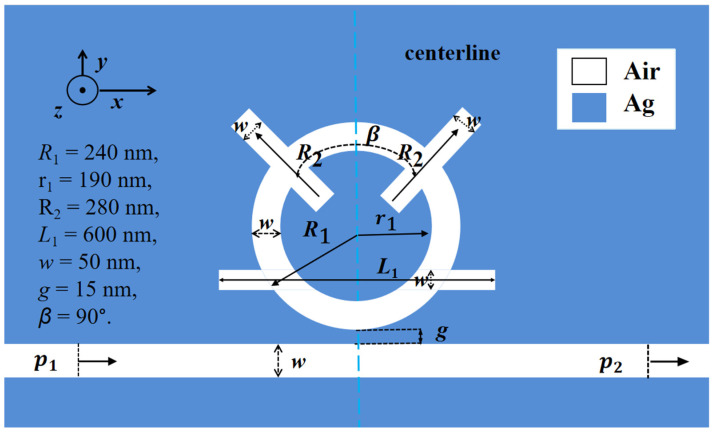
The 2D structural design diagram.

Figure 1 shows the geometric model of the coupling structure. It was built using COMSOL software. Since the structural thickness exceeds the optical wavelength, a 2D model is applied for simulation. The parameters for the coupled structure are set as follows:

- *R*_1_ and r_1_ are the external radius and inner radius of the CFSR;
- R_2_ is the length of the top stub on the CFSR;
- *L*_1_ is the length of the bottom stub on the CFSR;
- *w* is the width of the stubs;
- *β* is the angular separation between the top stubs;
- *g* is the gap between the waveguide and the resonator;
- *p*_1_ is optical input port;
- *p*_2_ is optical output port.

The silver and air used as dielectric fillers, along with their respective filling regions, are indicated in Figure 1.

To analyze the model, we first need to calculate the relative permittivity of the silver substrate [35]. For this calculation, the Debye–Drude model is given by:

(1) $$\varepsilon (\omega )=\varepsilon_{\infty }+\frac{\varepsilon_{s}-\varepsilon_{\infty }}{1+i\tau \omega }+\frac{\sigma }{i\omega \varepsilon_{0}}$$

Here, $\varepsilon_{\infty }=3.8344$ represents the relative permittivity at infinite frequency. $\varepsilon_{s}=-9530.5.\tau =7.35\times 10^{-15}\;s.\;\sigma =1.149\times 10^{-7}\;S/m$.

TM modal equation is given by [36]:

(2) $$tanh(k\omega )=-\frac{2k\alpha_{c}}{k^{2}+p^{2}\alpha_{c}}$$

Here, *k* is the wave vector. αc = [k02×(εin−εm)+k]12 and k0=2πλ0; $P=\varepsilon_{\mathrm{in}}/\varepsilon_{m},{\;\varepsilon }_{\mathrm{in}}\;\mathrm{and}\;\varepsilon_{m}\;$ are the dielectric constants of the insulator and the metal, respectively.

S and FOM are two key parameters for evaluating sensor performance. S quantifies the sensor’s response strength to refractive index changes, while FOM incorporates both response magnitude and spectral resolution, thereby directly determining detection limits and resolution. Although the quality factor (Q) describes resonance sharpness, it is inherently embedded within FOM. Focusing on FOM rather than Q alone allows for a more comprehensive evaluation and optimization of overall sensor performance. Their calculation formulas are introduced as follows [37]:

(3) S=∆λ∕∆n,

(4) FOM=S∕FWHM,

Here, ∆*λ* is the wavelength shift. ∆*n* is the corresponding change in refractive index. FWHM is the full width at half maximum.

We used COMSOL5.6 software to design the coupling structure in two dimensions and performed simulations to evaluate its performance. A fine mesh was used throughout the simulation process to ensure accuracy. Additionally, to make the simulation results more closely resemble reality, a Perfectly Matched Layer was applied in the boundary conditions. To excite the gap surface plasmon polariton mode within the metal–insulator–metal waveguide, we employed a transversely magnetically polarized plane wave as the excitation source in our simulations. This plane wave was incident perpendicularly at the model’s input port, with its magnetic field direction parallel to the device plane and perpendicular to the propagation direction, which is a prerequisite for exciting the desired waveguide mode. The light wave subsequently propagates along the waveguide axis, exiting through the output port after coupling with the resonator structure. At the simulation domain boundary, a perfectly matched layer was implemented to absorb the outgoing wave, thereby minimizing non-physical reflections. By scanning the incident light wavelength and calculating the output port transmittance, we obtained the system’s transmission spectrum. This configuration is equivalent to experimentally guiding laser light, ideally into the waveguide via end-face coupling or grating coupling, enabling us to focus on investigating the optical and sensing characteristics of the waveguide–resonator coupling structure itself.

**Figure 2:**
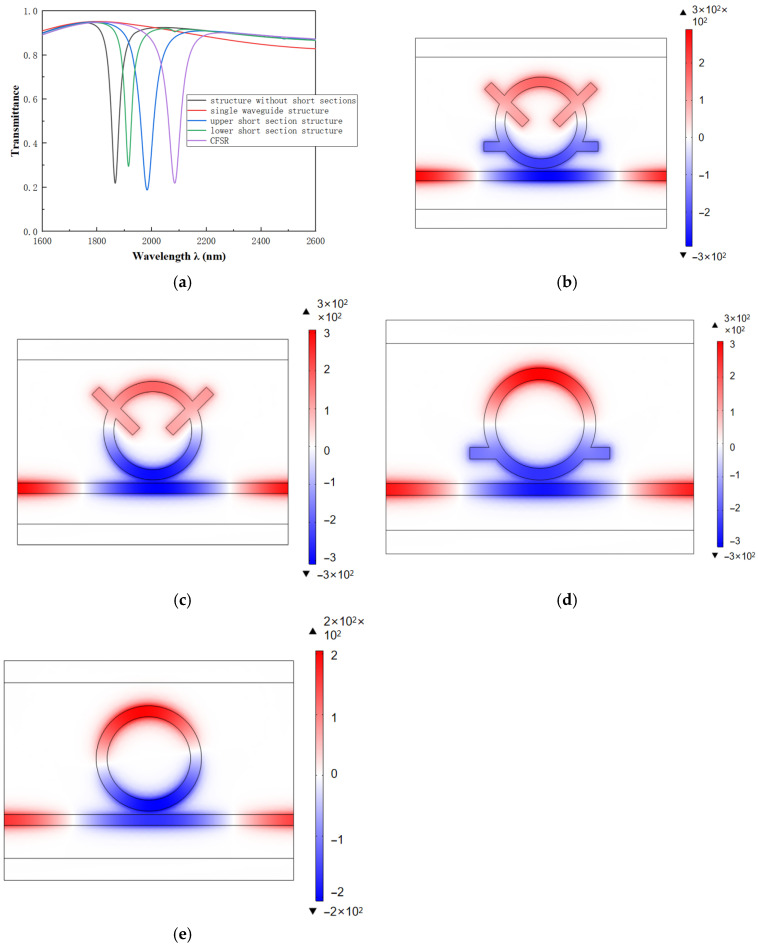
(**a**) Transmission spectra for different structures. (**b**) Magnetic field diagram of CFSR at λ = 2084 nm. (**c**) Magnetic field diagram of the upper short-section structure at λ = 1983 nm. (**d**) Magnetic field diagram of the lower short-section structure at λ = 1915 nm. (**e**) Magnetic field diagram of the non-short-section structure at λ = 1867 nm.

When evaluating the performance of the coupling structure, a comparative analysis was conducted to visualize the results and clarify the role of the stub structures. The following configurations were compared: a single waveguide, a ring resonator without stubs, a resonator with only the top stub, a resonator with only the bottom stub, and the complete CFSR. Figure 2 shows the transmission spectra obtained from this comparative analysis.

As shown in the figure, the spectral curve of the single waveguide structure exhibits a flat characteristic. Its transmittance is very high, indicating that it can be considered a broadband mode. The other structures show strongly asymmetric line shapes. This suggests the presence of distinct Fano resonance. This phenomenon is caused by the interference effect between adjacent broadband states and narrowband states. Meanwhile, as the structure changes, the resonance curve exhibits a significant red shift. This indicates a substantial improvement in the system’s sensitivity (S). Among the designs, the CFSR structure demonstrates the most significant enhancement. Next, we will explore which factors influence the performance of coupled structures.

## 3. Results and Discussion

**Figure 3:**
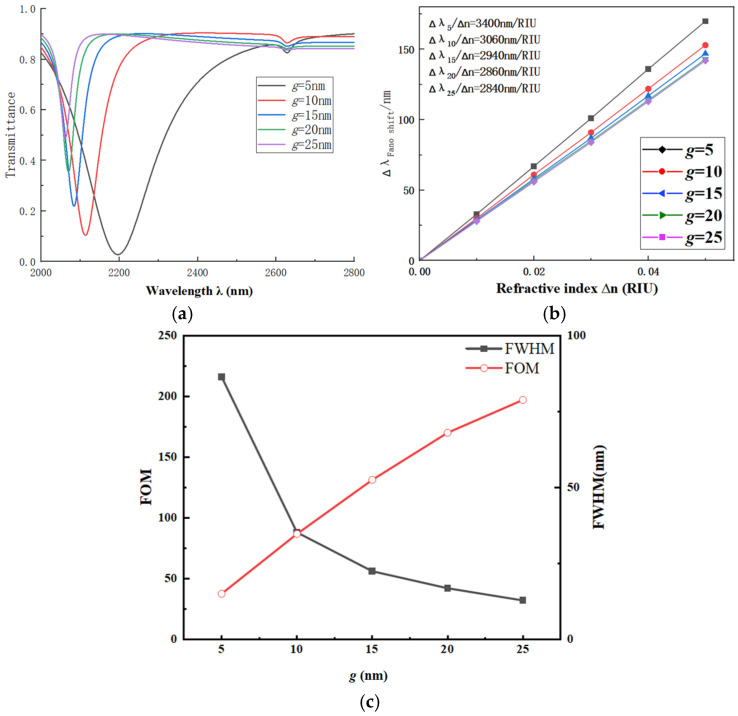
(**a**) Transmission spectra vs. gap *g*. (**b**) Fitted line vs. gap *g*. (**c**) Comparison of FWHM and FOM under different gaps *g*.

First, we discuss the impact of the coupling gap *g* on the performance of the CFSR structure. The gap *g* varies from 5 nm to 25 nm in increments of 5 nm. Other parameters are fixed as follows: *L*_1_ = 600 nm, *R*_1_ = 240 nm, *β* = 90°, R_2_ = 280 nm, *w* = 50 nm and r_1_ = 190 nm. The transmission spectrum with *g* as the variable is shown in Figure 3a. The fitted line for S variation is presented in Figure 3b.

Based on the variation observed in the transmission spectra, as the coupling distance *g* increases, a significant blue shift occurs in the spectrum. The corresponding S of the structure gradually decreases, dropping from 3400 nm/RIU to 2840 nm/RIU. Concurrently, the transmittance increases significantly, and the FWHM of the resonance narrows considerably. This leads to a further improvement in the system’s FOM. When selecting the value for the coupling distance *g*, comprehensive consideration was given to multiple factors. These factors included S, FOM, FWHM, and transmittance. Based on this analysis, *g* = 15 nm was determined to be the optimal value for the coupling distance.

**Figure 4:**
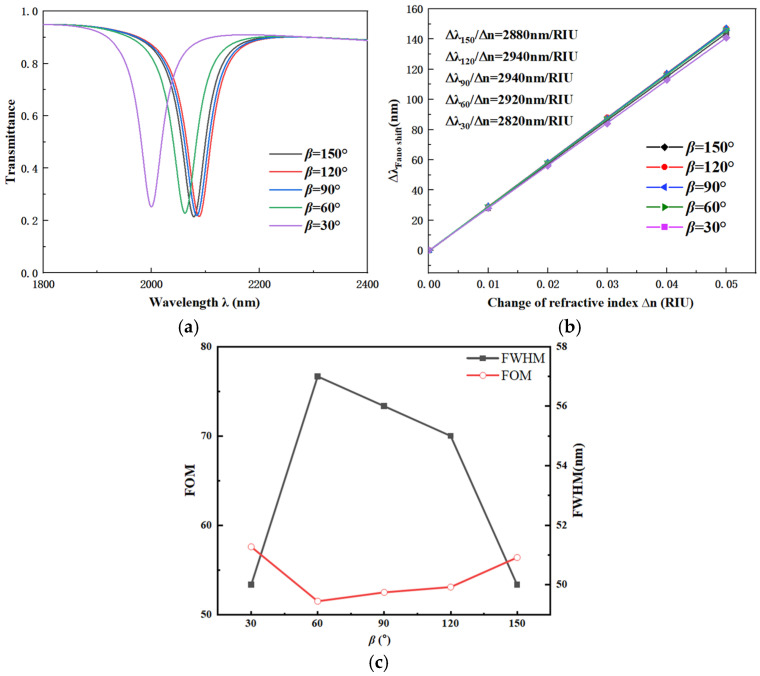
(**a**) Transmission spectra vs. angle *β*. (**b**) Fitted line vs. angle *β*. (**c**) Comparison of FWHM and FOM under different angles *β*.

Next, we investigate the influence of the angle *β* between the top stubs of the CFSR on its performance. The other parameters are chosen as below: *R*_1_ = 240 nm, r_1_ = 190 nm, R_2_ = 280 nm, *w* = 50 nm, and *g* = 15 nm. The value of the angle *β* is set to 150°, 120°, 90°, 60°, and 30°, respectively. The transmission spectra, with the stub angle *β* as the variable, are shown in Figure 4a. The corresponding fitted line for S variation is presented in Figure 4b.

According to the spectral curve variations, when the angle *β* decreases from 150° to 60°, the transmission spectra show minimal change. This indicates that variations within this range have a relatively small impact on the coupling performance of the CFSR. The S and FWHM are only weakly affected. However, when the angle *β* is further reduced to 30°, a distinct blue shift is observed in the transmission spectrum. Concurrently, the S decreases significantly, demonstrating that this degree of angular change has a substantial impact on performance. By comparing the spectral and fitting plots, the value of angle *β* was evaluated within the 150° to 60° range. A comprehensive comparison was conducted, considering the S, FOM, FWHM, and transmittance of the transmission spectra. Based on this analysis, the optimal value for the angle *β* was ultimately determined to be 90°.

**Figure 5:**
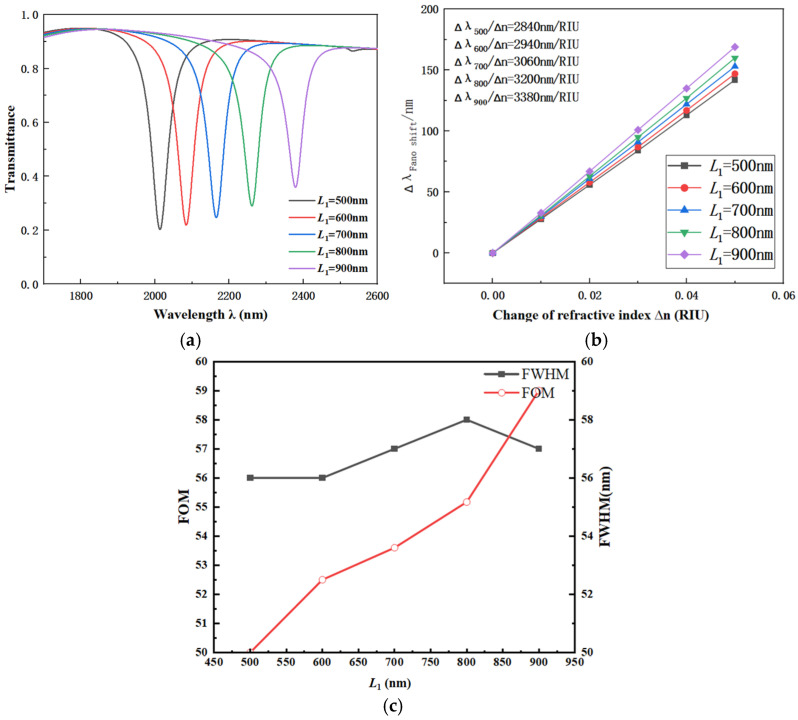
(**a**) Transmission spectra vs. length *L*_1_. (**b**) Fitted line vs. length *L*_1_. (**c**) Comparison of FWHM and FOM under different lengths *L*_1_.

Next, the influence of the bottom stub length *L*_1_ on the CFSR structure’s performance was tested. The other parameters were chosen as below: *R*_1_ = 240 nm, r_1_ = 190 nm, R_2_ = 280 nm, *w* = 50 nm, and *β* = 90°. The values for the bottom stub length *L*_1_ were set to 500 nm, 600 nm, 700 nm, 800 nm, and 900 nm, respectively. The transmission spectra, with the bottom stub length *L*_1_ as the variable, are shown in Figure 5a. The corresponding fitted line for S variation is presented in Figure 5b.

Based on the changes observed in the transmission spectra, as the bottom stub length *L*_1_ increased from 500 nm to 900 nm, a distinct red shift occurred in the transmission spectrum. This trend, combined with the variation shown in the sensitivity fitting plot, indicates a significant improvement in the sensitivity of the CFSR structure. Therefore, it can be concluded that variations in the length *L*_1_ of the bottom stub profoundly affect the performance of the CFSR. However, as the value of *L*_1_ increases, the transmittance of the corresponding structure also rises significantly. The increase in transmittance has a negative effect on the performance of the coupled structure. When selecting the value for *L*_1_, a comprehensive analysis was conducted, considering factors including S, the FOM, FWHM, and transmittance. To prevent the negative effect of excessively high transmittance on the CFSR’s performance, *L*_1_ = 600 nm was ultimately chosen as the optimal value.

**Figure 6:**
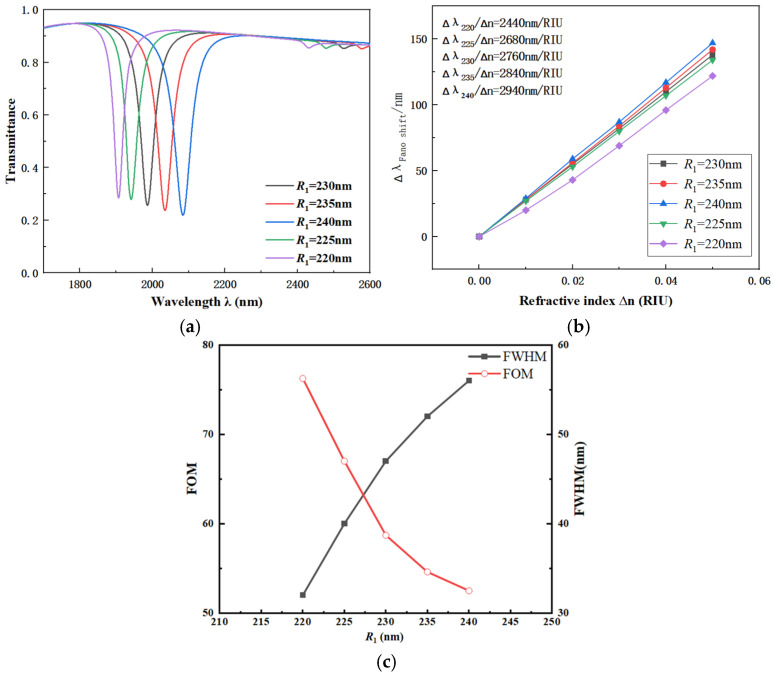
(**a**) Transmission spectra vs. radius *R*_1_. (**b**) Fitted line vs. radius *R*_1_. (**c**) Comparison of FWHM and FOM under different radii *R*_1_.

To further identify the factors affecting the coupling performance of the CFSR, the value of *R*_1_ was set to range from 220 to 240 nanometers. The other parameters were chosen as follows: *L*_1_ = 600 nm, r_1_ = 190 nm, R_2_ = 280 nm, *w* = 50 nm, and *β* = 90°. The transmission spectra, with the outer ring radius *R*_1_ as the variable, are shown in Figure 6a. The corresponding fitted line illustrating the S variation is presented in Figure 6b.

According to the spectral variation patterns shown in the figure, when *R*_1_ increases gradually from 220 nm to 240 nm, a significant red shift is observed in the spectrum. The S of the CFSR structure continues to increase, indicating that changes in *R*_1_ have a noticeable effect on the coupling performance. The FWHM of the system shows little variation, demonstrating that the system remains stable. By analyzing the S fitting curve in the figure, the corresponding S and FOM for each curve were calculated. Based on this analysis, *R*_1_ = 240 nm was selected as the optimal value.

**Figure 7:**
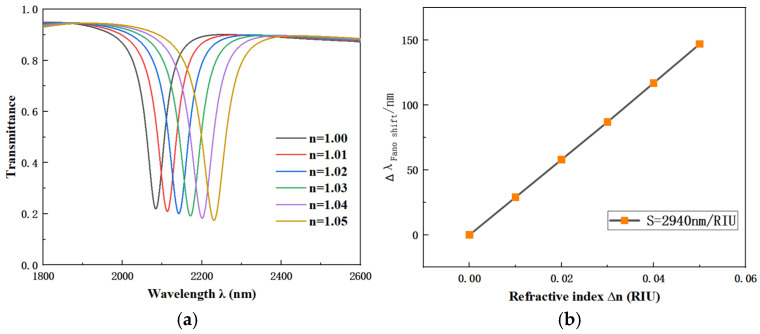
(**a**) Transmission spectra vs. refractive index *n*. (**b**) Fitted line vs. refractive index *n*.

After determining the optimal parameters, we investigated the effect of different refractive indices on the transmittance of the CFSR structure. The results are shown in Figure 7a. The corresponding S fitting curve is plotted in Figure 7b for a comprehensive performance analysis of the overall structure. The final performance metrics of the optimized CFSR structure are introduced as follows: S of 2940 nm/RIU, transmittance of 0.20, FWHM of 56 nm, and FOM of 52.5. Meanwhile, we compared several typical sensor designs to demonstrate the superiority of our proposed sensor, as shown in Table 1. Through comparisons of structural design, S, and FOM, the results indicate that our proposed sensor features a simpler structure, higher S, and a reasonable FOM.

## 4. Design and Applications

We propose the application of the designed CFSR structure in temperature sensing. Ethanol exhibits a high thermal–optical coefficient characteristic. This property enables the temperature detection required in this design. Furthermore, in this design, the permittivity of silver is much smaller than that of ethanol. Therefore, the effect of temperature on silver can be directly ignored. The correlation formula for ethanol refractive index and temperature is given as follows [42]:

(5) $$n=1.36-3.94\times 10^{-4}(T-T_{0})$$

Here, T_0_ is the standard temperature of 20 °C. *T* is the environmental temperature being detected. The formula relating temperature and S is

(6) $$S_{T}=∆\lambda /∆T$$

**Figure 8:**
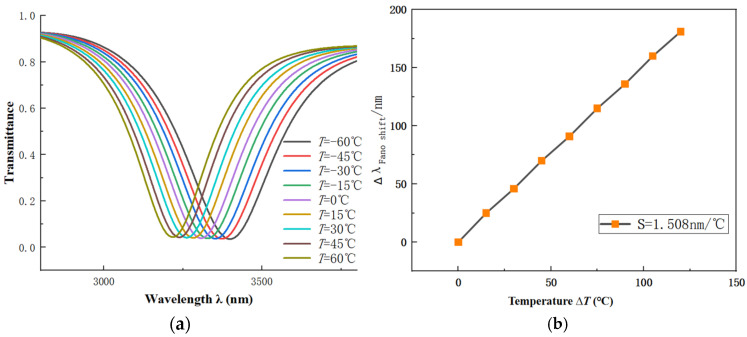
(**a**) Transmission spectra vs. temperature *T*. (**b**) Fitted line vs. temperature *T*.

The structural parameters are set as *R*_1_ = 240 nm, r_1_ = 190 nm, R_2_ = 280 nm, *L*_1_ = 600 nm, *w* = 50 nm, *g* = 15 nm, *β* = 90°. The temperature is set to 60, 45, 30, 15, 0, −15, −30, −45 and −60 °C. In Figure 8a, the temperature increases from −60 °C to 60 °C and the refractive index decreases due to its dependence on temperature. This results in a noticeable blue shift in the image. This trend is consistent with the spectral changes observed previously for refractive index variations. The basic shape of the spectra remains largely unchanged across different temperatures, indicating a strong correlation between the two. Additionally, the S fitting curve is plotted as in Figure 8b. This demonstrates the good performance with S of 1.508 nm/°C.

## 5. Conclusions

In this design, we propose a novel coupling structure. The structure consists of an MIM waveguide coupled with a circular four-stub resonator (CFSR). Simulation results demonstrate that it exhibits a pronounced Fano resonance phenomenon. Furthermore, this design also delves into which factors exert positive or negative effects on device performance. These parameters include the angle between the top stubs and the length of the bottom stub. The spectral variation under different refractive indices was also studied. After appropriate parameter optimization, the sensor performance achieved our design targets. The obtained S is 2940 nm/RIU, and the FOM is 52.5. Additionally, we explored the application of this sensor structure in temperature sensing. It also demonstrated excellent S performance, measured at 1.508 nm/°C.

## Figures and Tables

**Table 1:** Performance comparison between the proposed sensor and similar structures.

Reference	Year	Structure	Sensitivity (nm/RIU)	FOM
[38]	2020	Isosceles triangular cavity	1260	120
[39]	2021	MIM waveguide with two symmetrical rectangle baffles coupled with connected-concentric-double rings	2260	56.5
[40]	2022	A baffle waveguide and an r-shaped resonator	1333	5876
[41]	2024	A ring cavity internally connected to an h-shaped structure	2400	68.57
This work	2025	CFSR	2940	52.5

## Data Availability

The data used to support the findings of this study are available from the corresponding author upon reasonable request.

## References

[1] Bozhevolnyi S.I., Volkov V.S., Devaux E., Laluet J.-Y., Ebbesen T.W. (2006). Channel plasmon subwavelength waveguide components including interferometers and ring resonators. Nature.

[2] Kristensen A., Yang J.K.W., Bozhevolnyi S.I., Link S., Nordlander P., Halas N.J., Mortensen N.A. (2017). Plasmonic colour generation. Nat. Rev. Mater..

[3] Min B.K., Ostby E., Sorger V., Ulin-Avila E., Yang L., Zhang X., Vahala K. (2009). High-Q surface-plasmon-polariton whispering-gallery microcavity. Nature.

[4] Zhang J., Zhang L., Xu W. (2012). Surface plasmon polaritons: Physics and applications. J. Phys. D Appl. Phys..

[5] Zayats A.V., Smolyaninov I.I., Maradudin A.A. (2005). Nano-optics of surface plasmon polaritons. Phys. Rep..

[6] Pitarke J.M., Silkin V.M., Chulkov E.V., Echenique P.M. (2006). Theory of surface plasmons and surface-plasmon polaritons. Rep. Prog. Phys..

[7] Gladstone R.G., Dev S., Allen J., Allen M., Shvets G. (2022). Topological edge states of a long-range surface plasmon polariton at the telecommunication wavelength. Opt. Lett..

[8] Hovestädt M., Memczak H., Pleiner D., Zhang X., Rappich J., Bier F.F., Stöcklein W.F.M. (2014). Characterization of a new maleimido functionalization of gold for surface plasmon resonance spectroscopy. J. Mol. Recognit..

[9] Han X.X., Rodriguez R.S., Haynes C.L., Ozaki Y., Zhao B. (2021). Surface-enhanced Raman spectroscopy. Nat. Rev. Methods Primers.

[10] Moskovits M. (1985). Surface-enhanced spectroscopy. Rev. Mod. Phys..

[11] Islam M.R., Iftekher A.N.M., Hasan K.R., Nayen J., Bin Islam S., Khan M.I., Alam Chowdhury J., Mehjabin F., Islam M., Islam S. (2021). Design and analysis of a biochemical sensor based on surface plasmon resonance with ultra-high sensitivity. Plasmonics.

[12] Khajemiri Z., Hamidi S.M., Suwal O.K. (2018). Highly sensitive biochemical sensor based on nanostructured plasmonic interferometer. Opt. Commun..

[13] Cheben P., Halir R., Schmid J.H., Atwater H.A., Smith D.R. (2018). Subwavelength integrated photonics. Nature.

[14] Law M., Sirbuly D.J., Johnson J.C., Goldberger J., Saykally R.J., Yang P. (2004). Nanoribbon waveguides for subwavelength photonics integration. Science.

[15] Foley I.V.J.J., Ungaro C., Sun K., Gupta M.C., Gray S.K. (2015). Design of emitter structures based on resonant perfect absorption for thermophotovoltaic applications. Opt. Express.

[16] Ijaz S., Kang D., Rana A.S., Kim J., Chani M.T.S., Zubair M., Abbassi Q.H., Mehmood M.Q., Rho J. (2025). Metasurface Absorber–Emitter Pair-Integrated High-Efficiency Thermophotovoltaic System. ACS Photonics.

[17] Rui D., Zhang L.B., Ding H., Shen H., Wei Y., Su Y. (2025). Super-resolution imaging using surface plasmon resonance cavity lithography. Opt. Express.

[18] Gao P., Li X., Zhao Z., Ma X., Pu M., Wang C., Luo X. (2017). Pushing the plasmonic imaging nanolithography to nano-manufacturing. Opt. Commun..

[19] Pan U.N., Khandelia R., Sanpui P., Das S., Paul A., Chattopadhyay A. (2017). Protein-based multifunctional nanocarriers for imaging, photothermal therapy, and anticancer drug delivery. ACS Appl. Mater. Interfaces.

[20] Limonov M.F., Rybin M.V., Poddubny A.N., Kivshar Y.S. (2017). Fano resonances in photonics. Nat. Photonics.

[21] Miroshnichenko A.E., Flach S., Kivshar Y.S. (2010). Fano resonances in nanoscale structures. Rev. Mod. Phys..

[22] Fan P., Yu Z., Fan S., Brongersma M.L. (2014). Optical Fano resonance of an individual semiconductor nanostructure. Nat. Mater..

[23] Khanikaev A.B., Wu C., Shvets G. (2013). Fano-resonant metamaterials and their applications. Nanophotonics.

[24] Limonov M.F. (2021). Fano resonance for applications. Adv. Opt. Photonics.

[25] Kocabaş Ş.E., Veronis G., Miller D.A.B., Fan S. (2009). Modal analysis and coupling in metal-insulator-metal waveguides. Phys. Rev. B Condens. Matter Mater. Phys..

[26] Butt M.A. (2024). Plasmonic sensors based on a metal–insulator–metal waveguide—What do we know so far?. Sensors.

[27] Hill M.T., Marell M., Leong E.S.P., Smalbrugge B., Zhu Y., Sun M., van Veldhoven P.J., Geluk E.J., Karouta F., Oei Y.-S. (2009). Lasing in metal-insulator-metal sub-wavelength plasmonic waveguides. Opt. Express.

[28] Zegaar I., Hocini A., Harhouz A., Khedrouche D., Ben Salah H. (2024). An ultra-wideband bandstop plasmonic filter in mid-infrared band based on metal-insulator-metal waveguide coupled with an hexagonal resonator. J. Opt..

[29] Wu Q., Zhang Y., Qu D., Li C. (2022). MIM waveguide system with independently tunable double resonances and its application for two-parameter detection. Appl. Opt..

[30] Qi Y., Wu Q., Su M., Li H., Wang X. (2024). MIM waveguide refractive index sensor with graphene enhanced three-ring nested resonator Fano resonance. Phys. Scr..

[31] Huang S., Zhan C., Shan Y., Li Y., Chen W., Dai H., Cai K., Chen J., Cai W., Su Y. (2025). Ultra-sensitive hemoglobin sensing empowered by momentum-mismatch-driven quasi-bound states in the continuum. Phys. Lett. A.

[32] Tan T.C.W., Srivastava Y.K., Ako R.T., Wang W., Bhaskaran M., Sriram S., Al-Naib I., Plum E., Singh R. (2021). Active control of nanodielectric-induced THz quasi-BIC in flexible metasurfaces: A platform for modulation and sensing. Adv. Mater..

[33] Yusupov I., Filonov D., Bogdanov A., Ginzburg P., Rybin M.V., Slobozhanyuk A. (2021). Chipless wireless temperature sensor based on quasi-BIC resonance. Appl. Phys. Lett..

[34] Wu F., Wu J., Guo Z., Jiang H., Sun Y., Li Y., Ren J., Chen H. (2019). Giant enhancement of the Goos-Hänchen shift assisted by quasibound states in the continuum. Phys. Rev. Appl..

[35] Zhou G., Yan S., Chen L., Zhang X., Shen L., Liu P., Cui Y., Liu J., Li T., Ren Y. (2022). A nano refractive index sensing structure for monitoring hemoglobin concentration in human body. Nanomaterials.

[36] Hassan M.F., Sagor R.H., Amin M.R., Islam M.R., Alam M.S. (2021). Point of Care Detection of Blood Electrolytes and Glucose Utilizing Nano-Dot Enhanced Plasmonic Biosensor. IEEE Sens. J..

[37] Zhou F.Q., Qin F., Yi Z., Yao W.T., Liu Z.M., Wu X.W., Wu P.H. (2021). Ultra-wideband and wide-angle perfect solar energy absorber based on Ti nanorings surface plasmon resonance. Phys. Chem. Chem. Phys..

[38] Bazgir M., Jalalpour M., Zarrabi F.B., Arezoomand A.S. (2020). Design of an optical switch and sensor based on a MIM coupled waveguide using a DNA composite. J. Electron. Mater..

[39] Liu P., Yan S., Ren Y., Zhang X., Li T., Wu X., Shen L., Hua E. (2021). A MIM waveguide structure of a high-performance refractive index and temperature sensor based on Fano resonance. Appl. Sci..

[40] Rohimah S., Tian H., Wang J., Chen J., Li J., Liu X., Cui J., Xu Q., Hao Y. (2022). Fano resonance in the plasmonic structure of MIM waveguide with r-shaped resonator for refractive index sensor. Plasmonics.

[41] Chang S., Yan S., Liu F., Wang J., Cao Y., Huang B., Zhu C., Wu T., Ren Y. (2024). Nanorefractive index transducer using a ring cavity with an internal h-shaped cavity grounded on Fano resonance. PLoS ONE.

[42] Liu F., Yan S., Shen L., Liu P., Chen L., Zhang X., Liu G., Liu J., Li T., Ren Y. (2022). A nanoscale sensor based on a toroidal cavity with a built-in elliptical ring structure for temperature sensing application. Nanomaterials.

